# Clinical characterization of IRF2BPL mutation: Case series and review of the literature

**DOI:** 10.1097/MD.0000000000041078

**Published:** 2025-01-03

**Authors:** Xiaoxia Lou, Wenfeng Li, Mend Pang, Yanqiang Wang, Xinli Zhu, Jianhong Geng

**Affiliations:** a School of Clinical Medicine, Affiliated Hospital of Shandong Second Medical University, Weifang City, China; b Second Department of Neurology, Affiliated Hospital of Shandong Second Medical University, Weifang City, China; c Electrophysiology Room, Affiliated Hospital of Shandong Second Medical University, Weifang City, China.

**Keywords:** gene mutation, IRF2BPL, NEDAMSS, neurodevelopmental disorder

## Abstract

**Rationale:**

*IRF2BPL* is an intronless gene localized to chromosome 14q24.3 that encodes an interferon regulatory factor 2 binding-like protein. In this study, we reviewed the literature on mutations in the *IFR2BPL* gene. In addition, we report a case of Neurodevelopmental Disorder with Degeneration, Abnormal Movements, Loss of Speech and Seizures (NEDAMSS) caused by a mutation in the *IFR2BPL* gene. The aim of this report is to increase clinicians’ awareness of such clinical cases.

**Patient concerns:**

In this report, we discuss the case of a 15-year-old male patient. The patient started with epilepsy and dystonia and was treated with antiepileptic seizure medication, then he was admitted to our hospital for recurrent seizures of epilepsy and dystonia, and the diagnosis of NEDAMSS was confirmed by whole exome genetic testing.

**Diagnoses:**

Exome-wide genetic testing confirmed the diagnosis of NEADMSS due to *IRF2BPL*.

**Interventions:**

Exome-wide genetic testing reveals mutations in the *IFR2BPL* gene.

**Outcomes:**

Symptoms improved from before after antiepileptic seizure medication combined with drugs to improve dystonia.

**Lessons:**

We have come across a case of recurrent seizures of epilepsy and dystonia due to a mutation in the *IFR2BPL* gene for which no definitive treatment has been found. Recently, several studies have led to the discovery of a new drug for the treatment of NEDAMSS. CuII (atsm) (copper II diacetylbis(4-methylaminouracil)) (CuATSM) is a small-molecular-weight drug that can be administered orally and then used in the human body. The literature suggests that the underlying mechanism of CuATSM involves the restoration of mitochondrial function, including correction of the mitochondrial differentiation and mislocalization observed in cells from NEDAMSS patients, but extensive trials are needed to demonstrate its efficacy in *IFR2BPL*-related diseases.

## 1. Introduction

IRF2BPL is an intronless gene (also known as EAP1 or c14orf4) localized on chromosome 14q24.3. The gene encodes a 796 amino acid long interferon regulatory factor 2 binding-like protein, which is expressed in a variety of human tissues.^[1]^ In humans, IRF2BPL mutations cause a neurodevelopmental disorder known as Neurodevelopmental Disorder with Degeneration, Abnormal Movements, Loss of Speech and Seizures (NEDAMSS).^[2]^ Although its function is largely unknown, several clinical studies have suggested a possible role in neuronal development and homeostasis,^[3]^ gonadotropin-releasing hormone transcription,^[4]^ regulation of the ubiquitin–proteasome pathway,^[5]^ and ubiquitination and degradation of β-conjugated proteins in gastric cancer.^[6]^ To date, 31 patients with IRF2BPL mutations have been described in the literature.^[3,7–18]^ Here, we present a patient carrying a novel genetic variant of the IRF2BPL gene manifested as NEDAMSS. We analyzed the clinical presentation and treatment options of this patient and previously reported patients with IRF2BPL mutations in anticipation of better and faster identification and diagnosis of the disease.

## 2. Case presentation

The 15-year-old boy’s parents are both healthy. His main manifestations are epilepsy, autism, and dystonia, which progressively worsens. His family has no similar illnesses, half a month past the due date, cesarean section, weighed 9 pounds at birth, weak sucking ability, no history of asphyxia.

The boy reached the peak of his morbidity before the age of 15. He was diagnosed with epilepsy at birth. The main manifestation is has involuntary upper limb jerks and hand clumsiness, treatment with phenobarbital, sodium valproate, and levetiracetam. He learned to walk at the age of 2 and less verbal, but can say duplicate word, diagnosed with autism, rehabilitation was given with poor results. Involuntary jerks of both upper limbs disappears at about 9 years of age, discontinuation of “phenobarbital, sodium valproate, and levetiracetam.” At the age of 12, who appeared myoclonic jerks of the limbs, involuntary lifting of both upper limbs and stick out one’s lips, weakness of both lower limbs, the right lower limb is obvious, with abnormal posture of hands, feet and left shoulder, manifested by involuntary lifting of the hands, flexion of the fingers and toes of both hands, symptoms progressively worsen until they become unsteady and prone to falls. Completely unable to walk at age 14. Symptoms worsened again at about 14.5 years of age, accompanied by involuntary tilt back of the neck, have difficulty chewing, aggravated by external stimuli and/or stress, symptoms are worse at night, difficulty sleeping, occasional fecal incontinence. He was treated with oral baclofen tablets 3 times a day, but the results were not satisfactory, so he came to our hospital.

**Figure 1.**
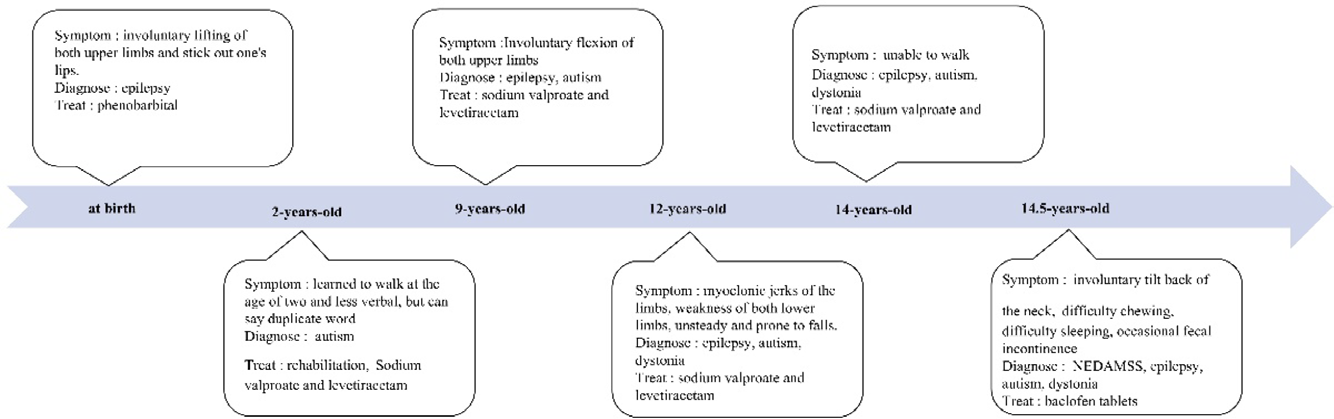
Patient onset time chart.

Physical examination of the nervous system shows, loss of speech, bilateral pupils are equal in size and circumference about 3 mm in diameter, sensitive to light reflection, limb flexion, involuntary limb movement, hypertonia of both hands and feet, inside left shoulder, the bilateral pathological signs were not elicited, and the physical examination of the residual nervous system did not cooperate (Fig. 1).

**Figure 2.**
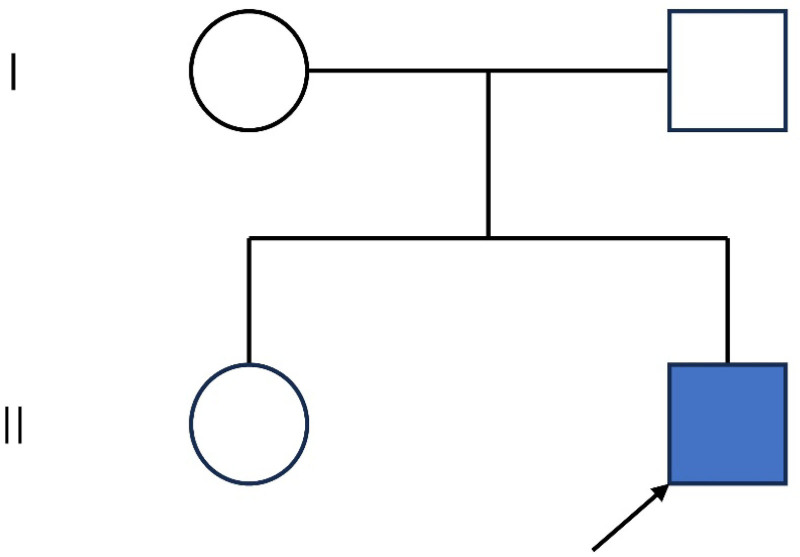
Proband’s family chart. II-2 proband; II-1 proband sister; I-1 proband mother; I-2 proband father.

Return of test results, blood routine: hemoglobin 115 g/L. Six items of blood coagulation: fibrinogen 1.39 g/L, thrombin time 21.5 s; thyroid function 3 items: thyrotropin 10.7 uIU/mL, the rest is normal; liver function: total protein 57.8 g/L, albumin 38 g/L; blood lipids: low density lipoprotein cholesterol 1.83 mmol/L; electrolytes: potassium 3.69 mmol/L, sodium 146 mmo1/L; no obvious abnormality was found in Hs-CRP, SAA, vitamin B12, folic acid, blood homocysteine, vitamin B1, and blood ammonia. Brain magnetic resonance imaging (MRI) showed mild brain atrophy-like changes. Two-hour video electroencephalogram (EEG) showed that during the awakening period of abnormal adolescents, the background fast wave activity increased, and the family members identified the event without seizure pattern. Genetic testing showed that NM_024496.4 (IRF2BPL): c.379C > T (p.Q127*) may be pathogenic, and the frequency of this variant in the gnomAD database is 0. This variant is nonsense and is predicted to result in the possible premature appearance of the termination codon, with multiple losses of function reported downstream of this locus, the patient’s father, mother, and sister did not carry the variant, considering the clinical relevance of this variant in the context of the patient’s clinical presentation, as a result he has been diagnosed with NEDAMSS, autism (Fig. 2). Sodium valproate oral solution 15 mL twice/day, levetiracetam tablets 0.125 g twice/day, clonazepam tablets 0.5 mg twice/day, baclofen tablets 15 mg in the morning, 10 mg in the afternoon and 10 mg at night were given. The patient did not have any further seizures and the dystonia improved over the previous period.

## 3. Literature review

Using PubMed as a search engine, the following keywords were used for the literature review: IRFBPL gene, epilepsy, dystonia. Our search identified more than 10 papers that provided clinical details of 31 patients harboring IRF2BPL mutations. We also found a recent paper written in English^[17]^ in which 11 new patients were reported; However, since we do not have access to the complete information contained in the latter, our review may be subject to some error.

The sample consisted of 32 patients (Table 1), 32 patients (100%) had developmental delays and/or motor/verbal deterioration of varying severity. Eighteen patients were reported to present with varying degrees of developmental delay. We did not find any correlation between age of onset and severity of degeneration at the time of examination. Other neurologic symptoms are present in the majority of patients, primarily reflecting involvement of the following neurologic systems, including epilepsy (26/32 patients), dystonia (20/32 patients), and ataxia (9/32 patients) (Table 2). Non-neurological problems were rare, included mild dysmorphic features (facial weakness, wider inter-nipple distance, joint laxity, etc) in 5/32 patients, cardiomyopathy in 1/32 patients,^[7–18]^ 1/32 patients presented with autism.

**Table 1:** Summary of case reports with mutation in the IRF2BPL gene.

Clinical features	Patient 1	Patient 2	Patient 3	Patient 4	Patient 5	Patient 6		Patient 7	Patient 8	Patient 9	Patient 10	Patient 11	Patient 12	Patient 13	Patient 14		Patient 15
onset age	14month	2 year-6-month	13 months	10-year-old	10 years	8.5years; deceased at 12 years		7 months	6 years	Deceased at 15 years	6 years	10 years	Deceased at 54 years	50 years + N3:PN3:P12	22 years		3.5 months
Gender	M	M	M	M	–	–		–	–	–	–	–	–	–	F		F
IRF2BPL variant	c.1171 C > T p.Arg391Cys	c.273_307del p.Ala92Thrfs*29	c.1157 C > T p.Thr386Met	C.562C > T (p.Arg188*)	c.373C > T (p. Gln125*)	c.519C > G (p.Tyr173*)		c.376C > T (p.Gln126*)	c.584G > T (p.Gly195Val) and c.514G > T(p.Gly172*)	c.562C > T (p. Arg188*)	c.379C > T (p.Gln127*)	c.376C > T (p.Gln126*)	c.581_599del, p. Gly194Alafs*12	c.584delG; p. (Gly195Alafs*17)	c.364C > T, p.Gln122Ter		c.232delG/p.V78Sfs*73
ACMG/Type of mutation	Likely	Pathogenic	Likely	Nonsense	Nonsense	Nonsense		Nonsense	Nonsense	Nonsense	Nonsense	Nonsense	Frameshi ft	Frameshi ft	Nonsense		Pathogenic
Seizure	Spasm combined with focal epilepsy,	Typical spasm. Minor seizures were observed after reduced anti-epileptic drugs	Spasm combined with focal epilepsy	no	Seizure; specifics not available	Seizure; specifics not available		Seizure; specifics not available	Seizure; specifics not available	None	Seizure; specifics not available	Seizure; specifics not available	None	None	Seizure; specifics not available		ES
Other neurological findings	None	None	Hypotonia (+);	Dysarthria (+); Hypotonia (+);	Dystonia (+); Hyperreflexia (+) Choreoathetosis (+);	Dystonia(+); ataxia(+); choreoathetosis (+); hyperreflexia(+);		Ataxia (+); tremor (+); dystonia (+); dysarthria (+);	Ataxia(+); dystonia (+); choreoathetosis (+);	Dystonia(+); dysarthria (+);	Dystonia (+)	Hypotonia (+); Ataxia (+); dystonia(+); dyskinesia(+); hyperreflexia(+);	Ataxia(+); dystonia(+);	Dystonia(+);	Myoclonic jerks(+); ataxia(+);		Hypotonia (+); Dysphagia(+);
Brain MRI	Bilateral frontal and temporal subdural spaces were widened	Normal	The subarachnoid space of the frontal and temporal pole is slightly wider	Normal	Generalized brain atrophy with possible increased iron deposits in deep gray matter	Diffuse atrophy		Normal	Not reported	(8 years) mild cerebellar atrophy, small cerebellum and “bulky” corpus callosum	(6 and 13 years) Normal; (15 years) thinning of corpus callosum	(34 years) global atrophy thinning of corpus callosum	Striatal atrophy	Not reported	Bilateral gliotic lesions.		Brain atrophy
EEG	Abnormal EEG of infants, hypsarrhythmia, frequent series of epileptic spasms and focal onset seizures were observed	Hypsarrhythmia, several isolated seizures were observed	Hypsarrhythmia. Frequent series of spasm, spasm mixed with wandering spasm were observed	Normal and did not show spike wave characteristics	No report	No report		No report	No report	No report	No report	No report	No report	No report	A standard electroencephalographic recording revealed diffuse epileptic form ab normalities and a photoparoxysmal response at 8–10 Hz photic stimulation.		Interictal period Continuous release of slow and sharp slow waves in the temporal regions bilaterally during wakefulness;
Treatment	Aminohexenoic acid treatment (1250 mg qd), seizure symptoms resolved within 2 weeks.	Corpus callosotomy to eliminate seizures. Epileptic spasms are effectively controlled	After 21 days of ACTH (25 IU daily) treatment, the spasms subsided but did not disappear. Therefore, topiramate (25 mg bid) was given. At 13 months of age, the patient still had 1 spastic episode per day	No report	No report	No report		No report	No report	No report	No report	No report	No report	No report	No report		Sodium valproate, topiramate, lamotrigine, aminocaproic acid’. ester, lamotrigine, aminocaproic acid
Clinical features	Patient 16	Patient 17	Patient 18	Patient 19	Patient 20	Patient 21	Patient 22	Patient 23	Patient 24	Patient 25	Patient 26	Patient 27	Patient 28	Patient 29	Patient 30	Patient 31	Our patient
Onset age	7 months	5 months	3.5 months	44 months	7 months	8.5 years	13 years	7 months	26 years	8 years	20 years	10 years	3 years	5 years	3 years 7months	6 months	9 years
Gender	F	M	F	F	F	M	F	M	F	M	M	F	M	M	F	F	M
IRF2BPL variant	c.244del/p.A82Pfs*70	c.1280C > T/p.L474F	c.1420C > T/p.S427L	c.1453-c.1455delTTC/p.F485del	c.283–308 (del/p.Ala95Thrfs*29)	c.519C > G (p.Tyr173*)	c.361C > T(p.Gln121*)	c.376C > T (p.Gln126*)	c.496G > T (p.Glu166*)	c.519C > G (p.Tyr173*)	c.562C > T (p.Arg188*)	c.962delC (p.Ala321Glufs*24)	c.2122delG (p. Ala708Profs*59)	c.2135_2136delGT (p.Leu713Serfs*56)	NA (p. Cys714Alafs*49)	c.2152delT (p.Cys718Alafs*48)	c.379C > T (p.Q127*)
ACMG/Type of mutation	Pathogenic	Likely	Likely	Likely	Pathogenic	No report	No report	No report	No report	No report	No report	No report	No report	No report	No report	No report	Likely
Seizure	Seizure; Specifics not available	Seizure; Specifics not available	Seizure; Specifics not available	Seizure; Specifics not available	Seizure; Specifics not available	Seizure; Specifics not available	Tonic–clonic-Seizure	Spasm, myoclonus; myoclony	seizures; myoclonus	No clinical seizure	no clinical seizure	2.5 months: tonic–clonic-seizures	No clinical seizure	Seizure; Specifics not available	No clinical seizure	Seizure; Specifics not available	Seizure; Specifics not available
Other neurological findings	Hypertonia (+); dysphagia (+);	Hypertonia (+)	Hypotonia (-); Dystonia (-); Ataxia (-);	Hypertonia(+)	Hypertonia(+)	Hypotonia (+); Dystonia (+); Ataxia (+);	Dystonia (-); Ataxia (-); Pyramidal syndrome	Dystonia (+); Ataxia (+);	Dystonia (NA); Ataxia (+);	Dystonia (-); Ataxia (+);	Dystonia (+); Ataxia (+); dysarthria (+);	Dystonia (-); Ataxia (+);	Dystonia (+); Ataxia (+); dysarthria(+);	Dystonia (-); Ataxia (-); Autism (+);	Dystonia (-); Ataxia (-); Dysphagia (+)	Dystonia (-); Ataxia (-); spasticity (+);	Dystonia (+); Ataxia (+)
Brain MRI	Normal	Normal	Delayed myelination	Normal	Delayed myelination	The absence of significant white matter anomalies on brain MRIs of the reported patients	The absence of significant white matter anomalies on brain MRIs of the reported patients	The absence of significant white matter anomalies on brain MRIs of the reported patients	The absence of significant white matter anomalies on brain MRIs of the reported patients	The absence of significant white matter anomalies on brain MRIs of the reported patients	The absence of significant white matter anomalies on brain MRIs of the reported patients	The absence of significant white matter anomalies on brain MRIs of the reported patients	The absence of significant white matter anomalies on brain MRIs of the reported patients	The absence of significant white matter anomalies on brain MRIs of the reported patients	The absence of significant white matter anomalies on brain MRIs of the reported patients	The absence of significant white matter anomalies on brain MRIs of the reported patients	Brain atrophy
EEG	Isolated or strings of spasmodic Spasms;	Isolated or strings of spasmodic seizures ‘spasms.	Isolated or series of spasmodic seizures Seizures; focal seizures	Isolated or strings of spasmodic seizures ‘spasms.	Isolated or strings of spasmodic seizures ‘spasms.	Points and diffuse waves predominating	Multifocal polyspikes and waves	Spikes and waves	Spikes and polyspikes	Frequent intermittent polymorphic theta slowing	Normal	Multifocal cerebral hyperexcitability with intermittent delta waves and sharp slow waves, hypsarrhythmia	Normal	Frequent centrotemporoparietal spikes alternating left and right, aggravated by sleep	NA	At seizures onset, hypsarrhythmia; at 1 year, diffuse discharge in the left hemisphere; at 2.2 years, diffuse discharges	During the awakening period of abnormal adolescents, the background fast wave activity increased, and the family members identified the event without seizure pattern.
Treatment	After enoic acid the spasmodic Clonic-seizures were significantly reduced, The use of topiramate, sodium valproate and levetiracetam was ineffective.	Oral aminocaproic acid seizure control	Previously, IV adrenocorticotropic hormone was ineffective and oral topiramate seizure control was achieved.	Oral topiramate and aminocaproic acid, IV ACTH day 7 seizure control	Seizure reduction with oral topiramate and aminocaproic acid, seizure control with IV ACTH	Topiramate, chlorpromazine Neurontin	Lamotrigine, levetiracetam, carbamazepine, clobazam	Chlorohexenoic acid CHCl3, hydrocortisone, and valproate 17 years: Sodium valproate, lamotrigine	Chlorpromazine, levetiracetam	Amantadine, L-dopamine, carbidopamine	Baclofen pump	Chlorohexenoic acid CHCl3, topiramate, chlorpromazine, phenobarbitone, CBZ (carbamazepine), valproate, zonisamid, rufinamid 7 years: vagal nerve stimulator	NA	NA	NA	VPA, LV, VG, TP, LM, hormonal treatment	Sodium valproate oral solution, levetiracetam tablets, clonazepam tablets, baclofen tablets

EEG = electroencephalogram, MRI = magnetic resonance imaging.

**Table 2:** Distribution of clinical symptoms in patients with (n. 26) or without (n. 6) magnetic resonance imaging abnormalities.

Clinical feature	Presence of MRI abnormalities (n. 26)	Lack of MRI abnormalities (n. 6)
Epilepsy (n. pts.)	17	5
Dystonia (n. pts.)	10	5
Ataxia (n. pts.)	6	1

MRI = magnetic resonance imaging.

Epilepsy manifested with multiple types of seizures, including myoclonic, infantile spasms, absences, generalized tonic–clonic-seizures, and focal seizures. Due to incomplete data we collected only 26 patients with perfected EEG, of which 18/26 patients had epileptic manifestations associated with EEG abnormalities. We could not observe any correlation between age of onset of epilepsy and seizures type. Several drugs have been used to treat the wide spectrum of epileptic manifestations that characterized the IRF2BPL gene mutation syndrome, including lamotrigine, carba mazepine, oxcarbazepine, topiramate, valproic acid, clonazepam, pirempanel, and high-dose paracetamol, etc. In our case, for example, we obtained good response with levetiracetam, valproic acid, benzoin resin, as it would be expected in myoclonic seizures. Thus, treatment of epileptic seizures in subjects carrying the IRF2BPL gene mutation did not appear to differ from that of subjects with other syndromes, even in the occurrence of drug resistance. Recently, some studies have found a new potential drug to treat NEDAMSS. CuII (atsm) (diacetyl bis (4-methylaminouric acid copper II)) (CuATSM) is a small molecular weight drug that can be used in human body after oral administration, which can cross the human blood–brain barrier. CuATSM is thought to play a neuroprotective role in areas affected by central nervous system diseases such as amyotrophic lateral sclerosis and Parkinson disease.^[19,20]^ The literature suggests that the underlying mechanism of CuATSM involves the recovery of mitochondrial function, including the correction of increased mitochondrial fractionation and dislocation observed in cells of patients with NEDAMSS.^[20]^

MRI abnormalities were detected in 23 patients and mainly consisted in focal or diffuse cortical/ subcortical atrophy, cerebellar atrophy, and thin ning of the corpus callosum. Other less frequent features were supratentorial T2/Flair hyperintensities, subarachnoid hemorrhage, putaminal atrophy, increased iron deposits in basal ganglia, thickening of corpus callosum.

Since the available information does not allow the assessment of the severity of neurological symptoms, we regard the number of neurological areas (epilepsy, dystonia, ataxia) related to stunting/ degeneration as the clinical severity of IRF2BPL-related diseases.

## 4. Discussion

IRF2BPL gene is located on the 14q24.3 chromosome encoding interferon regulatory factor 2 binding-like protein.^[1]^ The protein is expressed in a variety of human tissues, including the brain. Although its function is largely unknown, some preclinical and clinical studies have shown that it may play a role in neuronal development and balance in vivo, the transcription of gonadotropin-releasing hormone, the regulation of ubiquitin–proteasome pathway and the ubiquitin and degradation of β-catenin in gastric cancer.^[4]^ The 32 subjects collected here have a series of neurological manifestations of different severity. The phenotype demonstrates a complex and progressive neurological disorder, with progressive symptoms of abnormal movements, loss of speech, seizures/epileptic encephalopathies, neurodevelopmental delay, dystonia, ataxia, dysphagia/dysarthria, and general cerebellar and pyramidal signs. The disorder is also known as NEDAMSS.^[2,21]^ Cases of NEDAMSS often report dysarthria and abnormal movements, as seen in our cases. In our case, the patient developed severe dystonia in a short period of time until he was unable to walk on his own. Some studies have shown that IRF2BPL contains 2 highly conserved domains, the N-terminal and C-terminal zinc finger DNA binding domain and C3HC4 RING finger domain of IRF2BP, both of which are involved in transcriptional regulation.^[22,23]^ Studies have shown that patients with pathogenic mutations in the poly Q domain have a higher incidence of epilepsy than patients with mutations in other fields. Mutations located before the first PEST block are more likely to lead to non-epileptic features of dystonia and ataxia than mutations near the C-terminal.^[24]^ In summary, it is further proved that IRF2BPL mutation can lead to a variety of nervous system manifestations and nonnervous system phenotypes, indicating that the gene may play an important role in development and neuronal homeostasis. Recent data show that IRF2BPL/Pits plays a role in down-regulating Wnt signal transduction in the nervous system.^[20]^ The first report to demonstrate that it plays a key role in the central nervous system shows that knockout of Drosophila lineal homologous gene Pits leads to neurodegeneration.^[3]^ In the central nervous system, neurons are supported by a variety of glial cells. Astrocytes are the most abundant cell types in the central nervous system. They regulate the extracellular environment of neurons and regulate synaptic transmission and plasticity, which are important for neuronal signal transmission and development and often play an important role in diseases. Some experiments have proved that part of the full-length IRF2BPL is mislocated on the cytoplasm in astrocytes of NEDAMSS patients, which may play a role in the pathogenesis of the disease.^[22]^ Further functional studies are needed to determine whether the loss of IRF2BPL transcriptional regulatory activity directly leads to differential expression of neurodevelopmental genes.

## 5. Conclusion

We describe a Chinese patient with NEDAMSS and autism due to nonsense IRF2BPL mutation. The imaging and EEG findings are intact. As previously reported, the clinical features of IRF2BPL mutation include various epileptic syndrome, growth retardation, brain atrophy shown by neuroimaging and abnormal EEG.^[4–6]^ It has been reported that turning off the distal gene mutation of the C-terminal RING finger of IRF2BPL protein may lead to more severe phenotype.^[9]^ At the same time, some studies have shown that IRF2BPL/Pits plays a role in down-regulating Wnt signal transduction in the nervous system.^[20]^ However, our patients are characterized by NEDAMSS and autism, and the mutation may be farther away from the distal end. In addition, it may be related to the expression level of IRF2BPL gene. At the same time, we also found a new potential candidate for the treatment of NEDAMSS-CuII (atsm).^[22]^ In short, the evaluation of the genetic pattern, age of onset and affected nervous system areas of IRF2BPL mutation cases may contribute to a better understanding of the genes that should be included in the detection panel, so that the new generation sequencing and whole exome sequencing will be targeted as further testing items.

## Author contributions

**Funding acquisition:** Jianhong Geng, Yanqiang Wang.

**Resources:** Jianhong Geng.

**Writing – original draft:** Xiaoxia Lou, Jianhong Geng.

**Writing – review & editing:** Wenfeng Li, Meng Pang, Xinli Zhu.
